# Practical Chemistry and Pharmacy

**Published:** 1894-07

**Authors:** 

**Affiliations:** LaGrange, Ga.


### PRACTICAL CHEMISTRY AND PHARMACY.

EDITED BY HENRY R. SLACK, M.D., PH.M., LAGRANGE, GA.

### ARTIFICIAL CITRIC ACID FROM GRAPE SUGAR.

The discovery that artificial citric acid may be produced from grape sugar through the medium of a peculiar ferment has been mentioned before (ATLANTA MEDICAL AND SURGICAL JOURNAL, June, 1894, page 245), and we now learn that the patented process of C. Wehmer has already found practical application in two factories established in Alsace-Lorraine. From the *Suddentsche Apotheker-Zeitung* we take the following details: The citric acid fungi or myedia are obtained by exposing for several days a five to ten per cent. solution of cane or grape sugar, and then transferring the ferment, for the purpose of procuring pure cultures, to a sterilized culture medicine which preferably contains about one or two per cent. of citric acid. The pure citric acid ferment thus obtained is then added to the tock solution, consisting of a mixture of cane sugar, grape sugar, maltose and dextrin of from three to thirty per cent. concentration, and which may contain the usual nutritive phosphates and nitrates. Fermentation is allowed to proceed at ordinary temperature for one or two weeks, with free access of atmospheric oxygen, the process resembling vinegar fermentation. Free citric acid to the amount of ten per cent. may be thus obtained, but if the process is not interrupted the product will ultimately be destroyed. The conversion of the glucose may be represented by the following formula: $C_6 H_{12} O_6 + O_3 = C_6 H_8 O_7 + 2H_2O$.—*Western Druggist.*

---

### STERILIZING WATER FOR DOMESTIC PURPOSES.

Boiling water for drinking purposes is always a troublesome procedure. Considerable time is required before it is fit for use, and it has a flat, mawkish taste. Among the many experiments made and plans carried out in relation to this subject, Kettell's plan of purifying drinking water by the use of iron chloride has been found to be about the best, and his observations have been confirmed by Watts (*Chem. News*, 1893, No. 1768). He says that in case of a hard water, it suffices to add neutral ferric chloride until a perceptible precipitate is produced; and that in case of soft water the addition of a small quantity of lime water (or a dilute solution of sodium carbonate) will cause precipitation, which latter is promoted by vigorous stirring. The sediment is allowed to settle, and the clear water is drawn off for use; it may first be passed through an ordinary filter paper. The author ascertained that 1 to 1½ fl. oz (30–45 c. c.) of iron perchloride suffice to purify 100 gallons (3.8 hectolitres) of water. For ordinary domestic purposes, the official solution should be considerably diluted, about tenfold, and a teaspoonful of it be used to each gallon of water to be sterilized. Water prepared at night will be ready for use the next morning.—*M. Pharm. Journal.*

### SOLNINE.

In a paper presented to the Cincinnati Section of the American Chemical Society, Professor J. N. Lloyd describes the isolation by him of an alkaloid from solanum carolinense popularly known as "poison or ground potato," "horse nettle" and "tread soft," which has long been used among the negroes of the South as a domestic remedy for "fits" and convulsions. The weed alkaloid to which he has given the name of solnine occurs in white, brilliant crystals of tabular form, which are practically insoluble in water and dilute ammonia. They dissolve freely in dilute acids, forming very soluble salts that are acid and bitter, leaving a persistent tingling sensation on the tongue. The salts have not yet been crystallized. Solnine is very soluble in cold chloroform and in boiling alcohol; from the latter it separates on cooling in large needle-like crystals, resembling hydrastine. It is precipitated in minute crystals from alcoholic solution by the addition of water. It yields ammonia on heating with caustic potash.—*Druggist Cir. and Chem. Gaz.*

### GUAIACOL EXTERNALLY AS AN ANTIPYRETIC.

Dr. S. Sciolla, of Genoa, the introducer of the endermic application of guaiacol as an antipyretic, used undiluted liquid guaiacol and paints this on the skin in different parts of the body (generally the extremities or the abdomen) in doses of two to ten cubic centimeters (one-half to two and a half fluid drachms), and then covers the part with a sheet of gutta-percha tissue or some other impermeable material. The antithermic effect of this treatment is said to be energetic—the temperature often falling to normal within a few hours. Several applications of guaiacol may be made daily, Sciolla's largest daily dose having been thirty grammes (one fluid ounce); it is advisable, however, not to have the first dose exceed two cubic centimeters (one-half fluid drachm).—*M. M. Rep. and Phar. Jour.*

---

### ANTIDOTE FOR CARBOLIC ACID POISONING.

Schobert recommends saccharate of lime as an antidote in phenol poisoning, as long as the poison is still in the stomach. After it has passed into the intestines, sodium sulphate is the proper antidote. The saccharate of lime is proposed as follows:

| | PARTS |
|---|---|
| Fresh quick lime | 15 |
| Sugar | 25 |
| Water | 1,000 |

The solution thus prepared, contains five per cent. of calcium hydroxide. This preparation is also a good antidote in oxalic acid poisoning.—*Pharm. Zeit.*

---

### INNOCUOUSNESS OF SALICYLIC ACID.

It has frequently been asserted that salicylic acid is injurious to gastric digestion. Dr. Perizoldt read a paper before the Pharmacological Section of the Congress of the German Naturalists and Physicians, in which he claimed to have exhaustively investigated the effects exercised upon the digestive activity of the stomach by various substances; and he proved that salicylic acid does not possess the slightest influence on the digestion of food, taken in the small doses used as a preservative, even when ingested with food, continuously for a long time.

